# *“I decided to participate….because I saw it as benefiting our community and families”*: a qualitative study of lay providers’ experiences with delivering an evidence-based mental health intervention for families in Uganda

**DOI:** 10.1186/s13033-023-00593-8

**Published:** 2023-08-21

**Authors:** Ozge Sensoy Bahar, William Byansi, Josephine Nabayinda, Joshua Kiyingi, Phionah Namatovu, Fithi Embaye, Mary M. McKay, Kimberly Hoagwood, Fred M. Ssewamala

**Affiliations:** 1 Brown School, Washington University in St. Louis, One Brookings Drive, St. Louis, MO 63130 USA; 2 International Center for Child Health and Development, Brown School, Washington University in St. Louis, St. Louis, MO USA; 3 Boston College School of Social Work, Chestnut Hill, MA USA; 4 International Center for Child Health and Development Field Office, Masaka, Uganda; 5 Office of the Provost, Washington University in St. Louis, St. Louis, MO USA; 6 School of Medicine, New York University, New York, NY USA

**Keywords:** Task-shifting, Child and adolescent mental health, Sub-saharan Africa, Qualitative, Evidence-based interventions

## Abstract

**Background:**

Children and adolescents who live in resource-limited communities in sub-Saharan Africa (SSA) experience significant mental health problems, including behavioral problems. In SSA, one of the most significant impediments to expanding services is a scarcity of mental health specialists. Task-shifting can effectively solve the mental health care gap in low-resource settings, yet it is underutilized in child and adolescent mental health. Moreover, the experiences of lay providers are understudied in global mental health, despite their potential impact on intervention effectiveness. In this study, we examined the experiences of community health workers and parent peers with the task-shifting of an evidence-based family strengthening intervention in Uganda.

**Methods:**

As part of a larger randomized clinical trial, semi-structured in-depth interviews were conducted with 24 facilitators selected using stratified purposive sampling. Interviews explored their decision to participate in the program; experiences with the training; and experiences with intervention delivery. All interviews were conducted in Luganda (local language) and audio recorded. They were transcribed verbatim and translated into English. Thematic analysis was used to analyze the data.

**Results:**

Despite concerns around lack of previous experience and time commitment, facilitators reported high relevance of the intervention to the families in their communities as well as their own as a motivation to participate. They also identified financial incentives as a motivating factor. These two factors also ensured their attendance at the training. They were satisfied with the content and skills provided during the training and felt prepared to deliver the intervention. During intervention delivery, they enjoyed seeing the families engaged and participating actively in the sessions as well as observing positive changes in the families. Some challenges with family attendance and engagement were noted. The facilitators reported an increased sense of self-efficacy and competence over time; and expressed high satisfaction with supervision.

**Conclusion:**

Facilitators’ positive experiences point to the high acceptability and appropriateness of task-shifting this intervention in low-resource settings. As the global mental health field continues to be interested in task-shifting interventions to lay providers, successful examples should be studied so that evidence-based models can be put in place to support them through the process.

## Background

Children and adolescents who live in resource-limited communities in sub-Saharan Africa (SSA) experience significant mental health problems, including behavioral problems [1, 2]. Emotional and behavioral problems are the most reported (40.8%) across SSA countries [1, 2]. This is particularly concerning as children in SSA comprise half of the total regional population, yet current mental health services are severely under-equipped to meet their needs [3, 4]. In SSA, one of the most significant impediments to expanding mental health services is a scarcity of mental health specialists, despite growing demand [5].

Given the serious consequences of failing to intervene when disruptive behavior disorders (DBDs) emerge [6–8], it is of utmost importance that effective and scalable evidence-based interventions that recognize the unique cultural and contextual challenges are implemented. Although effective interventions for the treatment of DBDs in youth have been tested in similar high- poverty and high-stress communities in developed countries [9, 10], and they may be relevant for widespread dissemination in low and middle-income countries (LMICs), most of these evidence-based interventions have not been utilized in SSA countries.

Resource constraints in LMICs, including the dearth of mental health professionals, have prompted calls for innovative approaches to expand the provision of mental health services in community settings [11]. Particularly, task shifting is a strategy for shifting tasks from specialized workers to non-specialist or lay workers in low-resource settings [5, 12–14]. Scholars argue that in low-resource settings, substituting specialists with low-cost community health workers provides a solution to the mental health care gap in many nations [15]. Under the right conditions (e.g., political support, embedded into the community, appropriate training and coaching, remuneration and incentive systems), this approach can lead to significant health gains [16, 17].

Task-shifting strategies that leverage existing government-supported systems and require few new resources are necessary for resource-constrained settings [18]. For children and adolescents, these systems include Education and Health, with delivery by individuals (e.g., teachers, and community health workers), already part of these systems [18]. In Uganda, the focus of this study, caregivers are embedded in the education system via parent-teacher associations (PTAs). PTAs are an established institution in the educational system of Uganda and act as a liaison between the parents and the school, with the purpose to improve the teacher-parent relationship, provide volunteers to the school and participate in formulating school policies [19]. This existing structure can be leveraged to identify caregivers interested in training to deliver child and adolescent mental health interventions. In addition, government officials have identified Village Health Teams (VHTs) as existing workforce options within the Ministry of Health. VHTs serve as the community’s initial point of contact for health. VHTs are community health workers, and they are considered an integral part of the national health structure. Community health workers tend to be stable in communities, residing in the same community for many years [20], and have already been playing a substantial role in health care provision [21–23]. Yet, they have not been tapped into for the delivery of evidence-based mental health interventions, especially in the context of child and adolescent mental health. In fact, a recent systematic review of family-based youth mental health interventions by non-specialist providers in LMICs only identified 10 studies [13]. Most importantly, to our knowledge, no studies have examined the experiences of parent peers trained to deliver an evidence-based mental health intervention.

Despite the potential promise, the global mental health literature includes only a few empirical studies of lay counselor perspectives on the acceptability, feasibility, and/or appropriateness of delivering mental health interventions in the context of child and adolescent mental health interventions [18], and most importantly none of these studies are focused on parent peers as lay counselors. A recent qualitative systematic review of ten studies focused on the experiences of lay health workers trained in task-shifting psychological interventions showed that lay health workers were satisfied with training but wanted more robust supervision [24]. They thought that not enough time was given to training in understanding mental health problems. Finally, lay health workers grew in confidence, which in turn, impacted their personal relationships with others. Yet only one of these studies was conducted in sub-Saharan Africa and none of the studies focused on child and adolescent mental health. One exception is a recent qualitative study exploring lay counselors’ experiences with delivering a family-based child mental health intervention in Kenya [25]. This study included a range of lay providers (e.g., business owners, farmers, pastors), though none specified as parent peers, where the lay providers reported intrinsic motivation to become counselors and high self-efficacy after training. They also reported positive experiences in their roles. The main challenges included client engagement, balancing responsibilities, stress, and burnout. Finally, a quantitative evaluation of lay counselors’ (30 community health workers and 30 teachers) perspectives on the acceptability and feasibility of providing a trauma-focused cognitive behavioral therapy intervention for children in Kenya showed that lay counselors in both sectors perceived the intervention to be a satisfactory intervention, feasible in their respective settings and that delivering the intervention was appropriate to their role and sector’s (health and education) approach to helping children [18].

Lay workforce’s perspectives and experiences delivering mental health care in their systems have implications for the potential for scale-up and sustainment as well as any needed support. Yet, they are understudied, especially in the context of Uganda. In this study, we examined lay providers’ experiences -uniquely including community health workers and parent peers- with preparing for and delivering an evidence-based multiple family group (MFG) intervention, called Amaka Amasanyufu (Happy Families in Luganda, the local language in the study area), in southwestern Uganda. Specifically, we explored: (1) facilitators’ motivations and initial concerns about program participation; (2) facilitators’ experiences with the training; (3) facilitators’ experience with intervention delivery to better understand the appropriateness and acceptability of task-shifting this intervention to parent peers and community health workers in Uganda.

### Amaka Amasanyufu intervention

The Amaka Amansanyufu intervention was culturally adapted [26] from an evidence-based MFG intervention titled ‘*4Rs and 2Rs*’ designed to strengthen families with children experiencing behavioral challenges in the United States [27]. Working closely with community stakeholders, we drew upon the Theory of Triadic Influence [TTI; 28] and the adaptation coding framework developed by Stirman, Miller, Toder, and Calloway [29] to guide the cultural and contextual adaptation process. The adaptation was conducted in close collaboration with community stakeholders. Adjustments to intervention delivery and content were made while maintaining the core principles of the intervention: 4 Rs (Rules, Responsibility, Relationships, and Respectful Communication) and 2Ss (Stress and Social Support) (please refer to #26 for more details on the intervention adaptation).

The multiple family groups involved 10 to 20 families, with at least two generations of a family present in each session, including caregivers/guardians, siblings, or other extended family members. Lasting between 60 and 90 min, sessions involved group discussions, role-plays, and other activities to foster support, learning, and interaction both within the family and among the families in the group [27]. While the intervention is specifically designed for children with DBDs [10, 20, 30], the team included families with children in the same age range who did not screen positive for DBDs in the intervention to avoid any discrimination [26], given the high level of stigma toward mental health problems in Uganda [31, 32].

The SMART Africa-Uganda scale-up study is the first to test the effectiveness and implementation of this culturally-adapted MFG intervention among children experiencing behavioral challenges and their caregivers in Uganda. Results from the clinical trial have shown that the intervention was efficacious in reducing child behavioral and mental health problems as well as improving caregiver mental health among Ugandan children [33–35].

### Recruitment and training of Amaka Amasanyufu facilitators

Delivered in school settings, the Amaka Amasanyufu intervention incorporated task-shifting through utilizing community health workers and parent peers as facilitators of the sessions. The Amaka Amasanyufu sessions were facilitated by two parent peers or two community health workers depending on the study condition.

The research team worked closely with the parent-teacher association (PTA) in schools to identify potential parent peers and the Ministry of Health’s list of Village Health Teams within the districts where the schools were located to select the community health workers. A total of 95 facilitators (48 parent peers and 47 community health workers) were recruited, trained and delivered the intervention sessions. All facilitators signed a consent form. While community health workers recruited for the study were registered with the Ministry of Health and had prior training in delivering health and psychosocial services in communities, parents recruited in the study via PTAs had no prior training in these areas.

The training for the facilitators was conducted separately for parent peers and community health workers. The training was delivered by team members already trained in the intervention by the PI who developed the original intervention, and focused both on content and facilitation skills. The sessions included the discussion of the intervention’s six main components (4Rs and 2Ss); overview of the five main parts in each session and importance of following the same structure; and practice of each session through role plays. Specifically, facilitators paired up to prepare and deliver a 45-minute mock session during which the remaining facilitators and the SMART Africa trainers acted as children and caregivers. Upon the completion of the mock session, feedback was provided to the leading facilitators.

Upon completion of the training, the facilitators completed the Knowledge Skills and Attitude Test (KSAT) [36] to ensure that they mastered the content (a score of 85% or above was required). Certificates for completion of the Amaka Amasanyufu training were also awarded to the facilitators.

During intervention delivery, the SMART Africa trainers were present to observe all sessions and provided feedback to facilitators upon completion of the session. At the end of each session, facilitators, as well as caregivers, filled out a fidelity checklist. In addition, research assistants trained in using fidelity tools conducted independent fidelity observations for 25% of the sessions. The fidelity checklists tapped multiple dimensions of an MFG session, including presence and quality of facilitation related to the following: (1) session content; (2) group discussion; (3) use of activities to foster information exchange across families; (4) practice activities within families; (5) summary of learning; (6) explanation of homework; and (7) summary of family strengths.

## Methods

### Study overview

Data for this study comes from the scale-up longitudinal experimental study, referred to as the SMART Africa-Uganda study funded by the National Institute of Mental Health. The study used a mixed methods hybrid type II effectiveness-implementation design [37, 38] to concurrently test the effectiveness and examine the implementation of the Amaka Amasanyufu intervention. The SMART Africa-Uganda study was designed as a three-arm cluster randomized controlled trial conducted in 26 public primary schools in five districts in the greater Masaka region in southwestern Uganda [20]. Entire schools were randomized to one of three study conditions: (1) Control condition receiving bolstered standard of care (children received usual care consisting of mental health care support literature, reinforced with school support materials, including school notebooks and textbooks (*n* = 10 schools); (2) Amaka Amasanyufu delivered by parent peers (*n* = 8 schools); and (3) Amaka Amasanyufu delivered by community health workers (*n* = 8 schools).

The qualitative study was nested within the larger randomized clinical trial to provide a more nuanced and complete understanding of community health workers’ and parent peers’ experiences with the task-shifting of the intervention, including their perspectives on the training they received as well as their experiences delivering the intervention. Specifically, we used a sequential explanatory design [39] where the quantitative data was used to determine the sampling for the qualitative study that will ensure the representation of potentially varying experiences of facilitators (see methods section).

### Ethical considerations

All study procedures were approved by Washington University in St. Louis Review Board (#2016011088) and the in-country local IRBs in Uganda: Uganda Virus Research Institute (GC/127/16/05/555), and the Uganda National Council of Science and Technology (SS4090). The study was also overseen by the Data Safety and Monitoring Board at the National Institute of Mental Health. Written consent was obtained from all facilitators in the study.

### Participant selection for the qualitative interviews

As part of the larger study, a total of 95 facilitators (48 parent peers and 47 community health care workers) were recruited and trained and delivered the MFG sessions in 16 schools (8 treatment 1 and 8 treatment 2 schools). Of these 70.5% (n = 67) were females and 28% (n = 28) were males. All facilitators completed quantitative measures on sustainability (adapted program sustainability measure) [40], barriers to implementation (adapted implementation and feasibility measure) [36], group processes and perceived impact of the intervention on families (Facilitator measure adapted from Metropolitan Area Child Study measure) [36]. We used stratified purposive sampling to identify facilitators for the qualitative component of the study. Using the above-mentioned three measures completed by the facilitators during the 16-week assessments (post-intervention completion), we computed total scores to determine parent peers and community health workers with the lowest and highest perceived levels of success and grouped them in quartiles. We then randomly selected six facilitators from the lowest and six facilitators from the highest quartiles in each group, resulting in a sample size of 24 (12 parent peers and 12 community health workers) to ensure that facilitators with potentially varying experiences were represented. Facilitators were invited to participate in semi-structured interviews by the research team and all agreed to participate. Of the 24 facilitators, 54% (n = 13) were females and 46% (n = 11) were males.

### Qualitative data Collection

Face-to-face semi-structured in-depth interviews were conducted following intervention completion between September 2019 and January 2021. The overall interview guide was informed by the Practical, Robust Implementation, and Sustainability Model (PRISM) [41]. PRISM is a practical and comprehensive implementation framework that integrates aspects of diffusion of innovation, models for quality improvement, and Reach-Effectiveness-Adoption-Implementation-Maintenance (RE-AIM). PRISM provides a framework to study the interaction of interventions with the characteristics of multi-level contexts/factors which may influence uptake, implementation, integration, and in this case, youth outcomes (youth and adult caregiver response, provider/facilitator-level factors, community-level support) [20, 41]. For this study, we focused on the facilitator-level experiences related to the acceptability and appropriateness of task-shifting this evidence-based intervention as these may impact the effectiveness of the intervention on youth outcomes as outlined by the authors [20]. Specifically, we focused on: (1) facilitators’ motivations and initial concerns about program participation; (2) facilitators’ experiences with the training; (3) facilitators’ experience with intervention delivery.

Interviews were conducted in Luganda, the widely spoken language in the study region. Interview questions were translated (English to Luganda) and back-translated by two proficient team members. Questions were reviewed and revised by the study team that included research assistants and co-investigators fluent in both languages to ensure that they sounded natural and conversational, and conveyed the same meanings intended. Interviews were conducted by male and female research assistants fluent in both Luganda and English and trained extensively by researchers with qualitative research expertise. Interviews lasted between 43 and 146 min (mean = 96 min) and were conducted in a private place with only the research assistant and the participant present. All interviews were audio taped and field notes were taken upon completion of the interview.

### Data Analysis

Interviews were first transcribed verbatim and then translated from Luganda to English by research assistants fluent in both languages. Dedoose analytic software [42] was used for data analysis. We focused on facilitators’ (lay counselors) -in this case, parent peers and community health workers- experiences related to the task-shifting the delivery of an evidence-based intervention focused on children with behavioral challenges and their families in Uganda. We used thematic analysis [43–46] to use preexisting themes as well as identify subthemes that emerged from the data. Interview transcripts were initially read multiple times and independently coded by two research assistants under the supervision of the first author using sensitizing concepts informed by the existing literature and the content of the intervention as well as identifying emergent themes (open coding) [46]. Broader themes were broken down into smaller, more specific units until no further subcategory was necessary. Initial codes were discussed during team meetings and reorganized when necessary to create a final codebook that was used to code all transcripts.

The analysis, conducted by the first author who was not part of the data collection, compared and contrasted themes and categories to identify similarities, differences, and relationships among the findings. The findings were presented to a member of the research team who was not involved in the data analysis as well as an external qualitative researcher from Uganda not involved in the SMART Africa-Uganda study to discuss the plausibility of themes and related findings to further ensure the trustworthiness of the study [47].

Finally, the data were collected by both female and male research assistants from similar communities who interacted with the facilitators at the time of data collection. We ensured that the data collection team was different from the training and supervisory team so that previous interactions with facilitators would not impact the interviewing process. The first author, a white female from the United States, with experience in conducting studies in the study area, developed the interview guide in collaboration with the study PI and research team members from Uganda and with extensive research experience in the study area. The first author was not involved in data collection but analyzed the data and supervised the coding of the transcripts by two female research assistants from sub-Saharan Africa not involved in the original study design or data collection. In addition, the analysis and interpretation of the data were reviewed by another study PI (white, female) and a female researcher from Uganda who was not part of the study. The involvement of different study members from diverse backgrounds at various stages was essential in minimizing the potential bias in the interpretation of study findings.

## Findings

This study examined facilitators’ experiences with the task-shifting of an evidence-based intervention. Specifically, we inquired into facilitators’: (1) decision to participate in the program, including motivations and initial concerns; (2) experiences with the training, including content learned as well as barriers and facilitators to participation in the training; and (3) experiences with intervention delivery, including the positive and challenging aspects, perceived changes in facilitation over time, and experience with supervision (see Table 1).

**Table 1:** Themes and subthemes

Research question	Theme	Subthemes
**Decision to participate in the program**	***Motivation to participate***	• intervention’s focus on family well-being • intervention’s potential to resolve family conflict o within participating families o within facilitators’ families • monetary incentives for participation • promise to learn new knowledge and skills
	***Initial concerns***	• lack of confidence in facilitating a large group • time commitment
**Experiences with training**	***Content learned***	• facilitation skills o relevance to intervention delivery o wider applicability in professional life • six pillars of the intervention (4Rs and 2Ss) o relevance during intervention delivery o wider applicability in own’s family and community
	***Facilitators to attendance***	• transport refund • refreshments • content relevance • desire to honor the commitment made
	***Barriers to attendance***	• rainy season • conflict with other responsibilities
**Experiences with intervention delivery**	***Positive experiences***	• engaging with families • increased family participation in sessions • positive changes in families • newly gained position in the community
	***Challenges experienced***	• families’ timely arrival o reminder calls o rules about late arrivals • families’ participation during sessions • co-facilitating
	***Session facilitation over time***	• growing confidence • pride
	***Experience with supervision***	• constructive feedback • availability of ongoing support

### Decision to participate in the program

As part of their overall experience, the interviews explored how the facilitators made the decision to participate in the program, including both what motivated them and what their initial concerns -if any- were about the program.

**Motivation to participate.** As part of the effort to better understand the decision-making process, facilitators were asked what motivated them to participate in the Amaka Amasanyufu intervention as a facilitator. The facilitators’ major motivation for participation was the intervention’s focus on family well-being and its potential to resolve conflicts within families. This point was expressed by both parent peers and community health workers. The facilitators pointed to the relevance of improving family well-being within their communities. A community health worker (ID#1212, male) pointed to some of the challenges the families had been experiencing in their communities and saw this as an opportunity to become the “pillar” of this program in his own community.

*I decided to participate in this program because I saw it as benefiting our community and families. There has been a very big gap between the parent and child and so many families had lagged behind. I realized that I needed to be part of the facilitators who would help our community to bring about development….* (ID#:1212, community health worker, male).

A parent peer raised a similar point and went on to add that those families who received the intervention may become role models for other families.

*I realized that if families are trained and educated, they may be different from the rest of the families that may not have received such training. And for those that have been trained, they may serve as role models. That is why I desired to join.* (ID#: 1124, parent peer, female)

In addition to benefits at the family and community levels, both community health workers and parent peers identified the potential benefit of Amaka Amasanyufu to them as caregivers and their own families as a motivator for participation. A community health care worker (ID#: 1228, community health worker, female) shared that she wanted to participate because she was a parent and wanted to gain more experience on “*how to handle*” her children. Along similar lines, a parent peer mentioned that *“The phrase Amaka Amasanyufu made me feel that I would gain a lot of things which would help me run my family or even help my neighbors”* (ID#: 1109, parent peer, female).

Incentives were also mentioned as an additional motivation by some facilitators. The compensation that facilitators were to receive would not only allow them to cover their transportation but also other family-related expenses. Indeed, in addition to noting the potential of seeing the families being happy and without conflicts, one parent peer elaborated on the different ways the transport refund supported her family’s financial needs.

*We were told that we shall receive a transport refund which was helpful in supporting my family and also used it to buy books, pay school fees for my children and buy basic needs at home like sugar and soap. (ID#: 1140, parent peer, female)*

Similarly, a community health worker mentioned that in addition to the opportunity to teach children and parents in groups, the monetary incentive was a motivation as it *“could help us have something in a pocket, have a drink and a snack.”* (ID#: 1201, male).

A few facilitators also talked about the promise of learning new knowledge and skills. A parent peer (ID#: 1142, female) was excited to learn from the people she would be co-facilitating the sessions with as well as the trainers who would train them in delivering the sessions. For community health workers, that meant expanding their professional skillset. One community health worker (ID#: 1211, male), who had already been working as a parasocial worker and village health team member, saw it as an opportunity to *“make revisions on what I was earlier teaching in another project”*.

**Initial Concerns.** About half of the facilitators expressed some initial concerns about participation. The main concern, primarily noted by parent peers, was the lack of confidence to “stand in front of” and facilitate a big group. For most parent peers, this was the first time they would be presenting to a large group of people. A parent peer (ID#: 1127, female) discussed her initial concern about facilitating a group that included adults and how she could address their questions.

*First of all, how would I stand before people, because it was going to be my first time teaching, and these were adults. I was nervous about being asked questions by these people, and whether I would be able to respond to them. Those are some of the things that worried me most….*(ID#: 1127, parent peer, female)

She went on to add that the training conducted by the research team in preparation for intervention delivery﻿ eased her anxiety, specifically through the use of roleplays to practice.

*[During] the training, the team from ICHAD would give each one of us a chance to deliver a session before our fellow facilitators and by the time I left the blackboard, my fear was already gone.*

*A community health worker (ID#: 1252, male) also mentioned that “standing in front of a big group of people”* was a concern for her since she had never done it before. In addition, she was also concerned about whether she would have the time to deliver the sessions. This was a concern brought up by other facilitators as well. They thought that the length of the intervention and the required training would interfere with their personal and professional lives. For instance, one parent peer (ID#: 1142, female) mentioned her worry about managing her family responsibilities as a widow staying with her grandchildren. However, when it was time to deliver the sessions, she came up with a solution that would allow her to balance both.

*My other concern was about my family. I am a widow and I stay with some of my grandchildren. I was worried about managing my family alongside delivering the sessions. After thinking about it, I decided to come up with a timetable at home and I made sure that I wake up at 5:00 AM engage in all home chores and then go to deliver the sessions.* (ID#: 1142, parent peer, female)

Another community health worker was concerned about the program’s potential interference with his personal work. However, he was reassured that the day and time of the training would be agreed upon together as a group.

*Although some of us were hesitant at the beginning because we thought that the program would interfere with our personal work on some days, the team clarified this by informing that we shall be needed on certain days, particularly those we have agreed upon.* (ID#: 1252, community health worker, male)

One parent peer noted that in addition to the high passing grade required in the competency test following the training completion, she was also worried about the potential interference of the program with her teaching duties as the intervention sessions would be delivered during the school day.

*We had to teach both children and their parents during school time. I got so much worried, because I am a teacher in a certain school, and they couldn’t release us. So, I started contemplating how best I could facilitate the Amaka Amasanyufu and at the same time fulfill my duties at School. But…. the timetable was in my favor. I had no lesson between and after the time I was supposed to be at the training.* (ID#: 1124, parent peer, female)

### Experiences with training

As mentioned earlier, facilitators received training from the implementation team in preparation for the delivery of the Amaka Amasanyufu intervention. Facilitators discussed the content learned during the training as well as facilitators and barriers to attending the training sessions.

**Content learned.** Facilitators felt that both the content specific to the intervention and facilitation skills taught during the training were relevant and critical to successfully delivering the sessions. When asked about what the most helpful area covered during the training was, facilitators mainly identified the facilitation skills, including keeping participating families engaged, “*moving around the classroom while teaching*”, and respectfully communicating with both children and caregivers. For example, a parent peer identified the inclusion of everyone in the discussion and writing everyone’s response on the flipchart as some of the most helpful skills they learned during the training.

*The skill that was most helpful was to make sure that every person who attended the session participated such that we don’t give chance to only those who have raised their hands…. We also learned how important it is to write down every point that is being raised, such that it gives confidence to the other person who raised it*. (ID#: 1124, parent peer, female)

Along similar lines, the emphasis during the training that the families’ experiences should be respected as they were and that there were “*no right or wrong answers*” was welcomed by facilitators. A parent peer found this particularly empowering for families in the context of the study region where many caregivers are farmers and do not get the opportunity to speak up in large groups to express their opinions.

*The very first skill we learned was welcoming everyone’s answer. There was no right or wrong answer to make sure that people are not discouraged or do not think that they are despised or even think that what they have said had no sense. Given that most of our participants are farmers who spend most of their time in gardens, they hardly gather and meet with people. So to some of them, it was a wonderful experience to realize that they could speak among people and that their ideas are considered.* (ID#: 1127, female, parent peer)

A parent peer (ID#: 1124, female) who also raised the same point as an important takeaway from the training went on to add that this was a skill they implemented in their professional life as well.

*Being that I am a teacher by profession, I embraced the skill of having all answers right even when they are wrong, and I no longer tolerate children who laugh at their colleagues when they have raised a wrong answer. If they try to do it, I immediately stop them. (ID#: 1124, female, parent peer)*

Some participants also pointed to what they called the six pillars of the program, namely Rules, Relationships, Responsibility, Respectful communication (4Rs), and Stress and Social support (2Ss), as the most important content learned during the training. A parent peer (ID# 1124, female) discussed how they emphasized them in every session before discussing the main topic of the day, which *“made it easy for people to connect them”* to the topic at hand. A community health worker (ID#: 1211, male) pointed out that these pillars were not only helpful in delivering the session content but also in other aspects of their lives.

*The training helped me so much with the six pillars. I realized that these pillars were not only useful in the training but also are reflected in our day-to-day life, the communities where we live, churches, and in all leadership positions. I am really grateful for the expertise that was used to discover them.* (ID#: 1211, community health worker, male)

A community health worker (ID# 1257, female), along with some other facilitators, applied these pillars within her own family, especially when it came to formulating rules together with her children. This point was also brought up by a male parent peer. In addition to formulating rules as a family, a parent peer’s family started to also find solutions to the stressors encountered together as a family.

*The other part was about handling stress which gave me courage on how to handle stressors in my family. Before, I could handle my challenges alone and spend sleepless nights, but now I can share it with my children and we find a collective solution. There was also respectful communication. I realized that when a child calls you and you respond well, everything goes on well. Even involving your wife in planning for the money you have excited me more.* (ID# 1101, parent peer, male)

He went on to add that respectful communication and relationships, two of the pillars of the Amaka Amasanyufu intervention that he learned in the training, were helpful in his personal, professional, and family relationships.

*I learned respectful communication, and this has enabled me to be calm even when someone has done something bad unlike before when I could just speak mindlessly when am annoyed. I also learned to build relationships with my colleagues. With this skill, I have been able to give support to any person who is facing problems. Additionally, I learned to relate and work together as a family. Now we can sit as a family and plan what we are going to do the following day.* (ID# 1101, parent peer, male)

**Facilitators and barriers to attending the training sessions.** The interviews explored what made it easy or challenging for the facilitators to attend the training sessions. The most commonly noted factor was the transport refund that the facilitators received at the end of each training session. A parent peer (ID#: 1124, female) stated that this was unexpected for her since they were there as a “*student*” to learn and saw this as an expression of how much the trainers valued them.

*What motivated me to keep coming for the training was the fact that I was paid to come to the training. This surprised me because since I was born, it is the student who pays. But this time, it was me who was being trained but refunded with my transport for coming to the training. And on behalf of my colleagues, this was a motivating factor for us to attend the training. We saw it as an honor on our side. It encouraged us so much….*(ID#: 1124, parent peer, female)

The transport refund allowed the facilitators to use the transportation they needed to get to the venue without worrying about the cost implications. It also allowed them to return home to their other responsibilities in a timely manner. A parent peer facilitator (ID#: 1152, female) also mentioned that she was able to use part of the transport refund to buy household necessities.

*I also used to buy some items that were missing at home from the transport allowance we used to receive, which my husband liked so much. In fact, even when I slept off, he could wake me up so that I don’t miss the training*. (ID#: 1152, parent peer, female)

A few facilitators also appreciated the refreshments served during the training coupled with the transport refund, which according to a community health worker allowed their *“hearts to be at peace and pay attention to the objective of our coming”(* ID#: 1212, male).

In addition to being a motivation to enroll in the program in the first place, facilitators noted the relevance of the content to their communities and families as a factor that motivated them to continue attending the training sections. For instance, a parent peer shared that the first session made her “*like what I was learning and realize that our community would benefit in these sessions*” (ID#: 1142, female). She went on to add that the sessions made her feel “*smart*” in the community as her community members were impressed by her input informed by the session content. Similarly, a community health worker (ID#: 1217, female), discussed the relevance of the session contents both to her community and to her as a caregiver to her grandchildren.

*One of the things that made it easy for me to attend the training sessions is that I lived in a community that had so many problems, some of which would be addressed by the sessions. I decided to continue attending so that I can discover more solutions to the community problems. Given that I had grandchildren at home, I felt obliged to continue coming to the sessions so that can learn how to instill discipline in them as I was among the parents who had unruly children.* (ID#: 1217, community health worker, female)

For some, the motivation was that they had already committed to the program and wanted to honor their commitment. A parent peer (ID#: 1113, male) discussed that by willingly signing the consent document, she agreed to attend the training sessions, and hence made sure she did so.

*In the document we signed, it was very clear that if you are not willing to be a facilitator, there wasn’t a need for you to sign the document. And by signing the consent, you had agreed to all the terms, so in any way, I was supposed to attend the training, since I had already signed an agreement with them.* (ID#: 1113, parent peer, male)

A community health worker (ID#: 1201, male) had similar thoughts: *“I had accepted to work with the organization, so I had to attend the training”*. Hence, he adjusted his schedule accordingly so that he could attend to his other duties. This was also echoed by other facilitators. For instance, a parent peer (ID#:1142, female) created a timetable to ensure that she would organize her other responsibilities.

*The other aspect that made it easy for me to attend the training sessions was the timetable I formulated at home. Originally, I would just mix the activities, but after getting to know that I had other responsibilities, I came up with a timetable to enable me to distribute my roles well.* (ID#: 1142, parent peer, female)

When asked about what made it difficult for them to attend the training sessions, most of the facilitators reported that they did not experience any challenges. For those who did experience challenges, the most commonly shared challenge was the heavy rain. When the training was conducted during the rainy season, it made it difficult for people to attend the sessions given the poor and often muddy road conditions in the study area, causing them to be late to or miss the session.

A fewer number of facilitators mentioned other family/social responsibilities such as taking care of a sick family member or attending a colleague’s funeral as reasons for missing training sessions. Three facilitators mentioned that the length of the training sessions lasting between 9 am to 4 pm initially made it challenging for them. For one community health worker, it initially interfered with her doing home chores, which she addressed by *“I came up with a plan on how to manage other activities depending on the time I return home. When I developed the plan, everything started to become easy on my side*” (ID# 1217, female). A parent peer (ID #: 1127, female), appreciated the refreshments served but expressed that she needed something more substantial. Hence, she *“made sure that I always have rice in my house, which I would cook and pack in a small container to supplement the cake and soda I used to receive”.*

### Experiences with intervention delivery

As part of their experience with the program, we asked facilitators about their experiences with delivering the intervention. We explored aspects of the intervention delivery they enjoyed, the challenges they encountered, their perceptions of how their facilitation changed over time, and their supervision experience.

**Positive experiences with intervention delivery.** The most frequently mentioned aspect that the facilitators enjoyed during the intervention delivery was families’ engagement in the sessions. A parent peer (ID#: 1138, male) talked about how he enjoyed having children and adults in the sessions. He also appreciated the *“respect and trust”* he received from them as a facilitator.

*First of all, [what I liked] were the people who participated in the sessions including the young and adults. They were happy people and showed interest in the session; secondly was the respect and trust they showed us as their facilitators, which was so encouraging and filled me with happiness.* (ID#: 1138, parent peer, male)

A community health worker (ID#: 1203, male) appreciated families sharing their experiences and challenges.

*Sharing ideas was an exciting experience since people’s families have different challenges, those with problems put them forward and other family members could help in handling them. People gave more brilliant ideas than I expected.* (ID#: 1203, community health worker, male)

Another community health worker (ID#: 1237, female) also echoed similar sentiments and appreciated the good relationships they had with families and the school.

*What excited me most, we had a strong relationship with the families and the school where we used to converge for the sessions. They used to prepare desks for us. Guardians were very cooperative; they could respond positively anytime we contacted them.* (ID#: 1237, community health worker, female)

A parent peer (ID#: 1140, female) detailed the different ways the families, both children and caregivers, engaged in different sections of a session.

*Some of the things that excited me most was that time when I introduced the session and someone gives me an idea that relates well to the session….The other part was the let’s practice section when people accepted to voluntarily participate in the role plays and they would perfectly do it. Also, the let’s share section, they could share so much about their problems which we would solve as a group.* (ID#: 1140, parent peer, female)

The facilitators noted changes in families’ active participation as the sessions progressed. A community health worker (ID#: 1252, male) discussed how both caregivers and children were not engaged in the earlier sessions. However, as time went on, they participated more during the sessions and were timely, with minimal absence.

*At the beginning of the first sessions, both parents and children were not yet motivated about engaging in the sessions. Things seemed to be boring to them and they could hardly participate in class. However, as we reached session thirteen to the end, the sessions had become interesting, they were participating….We were also amazed by the way they could come early for the sessions, and with minimal absences.* (ID#: 1252, community health worker, male)

He went on to add that families reported positive changes in the way children behaved at home. A parent peer (ID#:1140, female) also mentioned that *“children would testify changes about their guardian and vice versa due to the sessions”.*

A few of the facilitators mentioned that they enjoyed their newly gained position in the community. A parent peer (ID#: 1106, female) shared* “I am proud that I became popular among other people, and wherever I go, people identify me as a facilitator.”* Similarly, a community health worker (ID#: 1237, female) also referred to getting a *“good public image”* as a facilitator.

**Challenges experienced.** The main challenges that the facilitators experienced revolved around ensuring families’ attendance and timely arrival to the sessions. Facilitators used a range of strategies, including reminder calls about the session and formulating rules about late arrivals. A parent peer (ID#: 1109, female) discussed how they decided that a person showing up late for the morning sessions would be required to attend the session offered in the afternoon.

*Some parents used to be late for the sessions, they could find us ahead which made them miss out on some important information. I reminded them of the importance of reaching the venue before the exact time of starting. I told them that if a person reports when the session has already started, that person will not attend the session except he/she will have to wait for the next session at 2 pm. That helped a lot because imagine coming when only twenty minutes past 9 am and you have to wait until 2 pm! They tried their best to report early.* (ID#: 1109, parent peer, female)

In a few cases, there was some overlap with the school schedule that made some children arrive late to the session. A parent peer (ID#: 1117, female) talked about the challenges of coordinating the start time with children’s break/lunch time or exams.

*Sometimes we had to wait for the children to be released during break time. This would take our time and there were times when they were doing examinations. So sometimes we could start facilitating when some children were still in class or still having lunch. We used to talk to the teachers, and they would request 5 to 10 additional minutes to enable children to get their porridge and lunch meal so that they can take it while sitting in the sessions. [And for exams] they used to ask us for more time, so we waited patiently*. (ID#: 1117, parent peer, female)

Another challenge facilitators struggled with at times was engaging participants during the sessions. They discussed how some children and caregivers would not want to volunteer a response. For instance, a community health worker (ID#: 1215, male) mentioned that when some caregivers chose to be “*active listeners*”, he chose not to push them for answers per what he was taught during the training.

*The other challenge was some of the parents who didn’t like to participate even though we liked it so much to involve all of them. These were only active listeners even without responding to a yes or no in class. With the training we received, we were advised to appreciate any response given by a guardian rather than pushing them for more responses.* (ID#: 1215, community health worker, male)

The strategy a parent peer used (ID#: 1128, female) in those instances was to remind them that every answer was right just as we had been told during the training.

A couple of the facilitators experienced challenges with their co-facilitator where they felt that their colleagues had not contributed to the sessions as they should have or that they went on too long. One community health worker (ID#: 1217, female) struggled with her co-facilitator who would interrupt her in the middle of her facilitation.

*I was challenged by my co-facilitator who used to interject me in the middle of my presentation. I didn’t like it because he couldn’t even warn you or request to give a hand but instead, interject whenever he felt like it. I tried to control my temper in front of the participants and let him complete his discussion and later continue with my presentation.* (ID#: 1217, community health worker, female)

A parent peer (ID#: 1152, female) also had problems with her co-facilitator who always went beyond the part that was agreed on.

*The challenge I encountered was about my co-facilitator who used to go beyond the section that she was supposed to stop at. Because we used to distribute the sections amongst ourselves, you could find that in some instances, my co-facilitator could go through my section, and I just remain seated as if I had not come to facilitate, which was challenging on my side.* (ID#: 1152, parent peer, female)

She went on to explain that this was because her co-facilitator did not attend the preparation session and instead prepared by herself. This situation was resolved by the training team that provided the necessary feedback to the co-facilitator.

**Session facilitation over time.** Facilitators were asked what they thought about their facilitation as the sessions progressed. Both parent peers and community health workers alike noted a positive change and reported being more confident in delivering the intervention as time went on. For instance, a parent peer (ID# 1138, male) discussed how he became more confident and excited around the third session.

*In the beginning, I had low self-esteem and couldn’t imagine that people would believe in me.But as I delivered up to the third session, I started to feel excitement and courage while I observed that people were believing in me. I also felt that I had gained experience. This is because, without experience, it is hard for people to comprehend what you are teaching them. (ID#: 1138, parent peer, male)*

Another parent peer (ID#: 1113, male) expressed a similar experience. He noted that he struggled at the beginning despite the training he received and added that it was probably harder for him because he was not trained as a teacher like some of the other facilitators. However, he felt more at ease with facilitating the sessions by the time the group reached the fifth session and felt he mastered it by the time he completed delivering all sixteen sessions.

*In the very beginning, it wasn’t easy even though I received training. It wasn’t until session five that I realized and understood the right way of delivering these sessions. But by the time I got to session five, I had grasped the gist of delivering the sessions. It is different with facilitators who are trained as professional teachers, but with farmers…. things were not easy. However, by the time I completed all sixteen sessions, I had become a master. But also, people liked how I was facilitating, and I believe from session five to the last, they understood all session topics.* (ID#: 1113, parent peer, male)

A community health worker (ID#: 1232, female) echoed the same sentiment and felt like she had become a lecturer at Makerere University, one of the top universities in the country, as time went on. She was so passionate about delivering the intervention that she and her colleagues went back to delivering more sessions.

*I had become like a real lecturer from Makerere University (laughs). From the training I received to the real facilitation, I had changed a lot. In fact, after completing the sessions, we went back to the school and resumed the sessions until we were called back for other activities.* (ID#: 1232, community health worker, female)

**Experience with supervision.** The feedback on the supervision that the facilitators received was positive. Facilitators appreciated both the guidance they received prior to the session delivery as well as upon its completion. A parent peer (ID#: 1127, female) described the process through which the supervising team provided support before each session.

*[The training team] was really supportive. They were available whenever we needed them. We used to meet the day before to review the sessions before we faced the group, but of course, there are those sections that were hard to understand and needed further guidance on them. This is where they could come in, to guide us and direct us on how best we could handle different sections of the session.* (ID#: 1127, parent peer, female)

A community health worker (ID#: 1203, male) echoed similar thoughts and said that these meetings served as a refresher before the sessions.

*They used to briefly guide us before the session begins, to remind us of what they trained us on. This helped a lot because we could deliver when we have a fresh memory of what transpired in that simple debrief.* (ID#: 1203, community health worker, male)

A community health worker (ID#: 1212, male) went on to add that the supervising team never told them how they thought the facilitators should conduct the session but instead would invite them to share any challenges they might have as they got ready to deliver.

While some facilitators were initially nervous about having the supervising team sit in the sessions and observe, they eventually realized that they were there to provide constructive feedback and not criticize them as illustrated by a parent peer (ID#: 1109, female) who said: “*You helped us so much indeed, at first I was hesitant on seeing them sitting in the class but later, I loved it.”* A community health worker (ID#: 1212, male) also had similar thoughts where he first was uncomfortable about having them in the session but eventually looked forward to receiving their feedback.

*We were so confident that in case something wasn’t clear, they would address it. In the beginning, we were afraid thinking that they had come to test us, but as sessions went on, we realized that they were part of us. Sometimes they could ask questions to make us understand that something wasn’t clear so that we could throw more light. This was helpful.* (ID#: 1212, community health worker, male).

Facilitators appreciated the respectful and subtle way that the supervising team provided feedback. A community health worker (ID#: 1215, male) discussed how the team guided them during the session where one of them would raise their hand like a participant and share a thought that would help the facilitator to get back on track.

*They helped us in so many different ways. They used to be part of the group and could seat at the back of the class but could actively participate and give in their opinions as participants, not as staff. In case of going astray amidst the session, they would raise a hand in disguise that they are contributing and then give you a point that would take you back on track and build on this.* (ID#: 1215, community health worker, male)

A parent peer (ID#: 1142, female) described a similar strategy used by the supervising team.

*They would raise a hand just like any other participant to give their opinion in disguise of correcting us. This helped me so much….They would pick up something in the manual to help you get back on track. I liked it so much and whenever I completed a session, I wanted to receive feedback from them*. (ID#1142, parent peer, female)

In addition, the facilitators appreciated the constructive feedback they received from the supervisory team during the debriefing at the end of each session. A community health worker (ID#: 1237, female) mentioned that the supervising team would not interfere during the session if an issue came up in the group but wait until the end of the session to debrief with the facilitators.

*There are times when there would be many arising questions during the sessions and people start whispering to one another. The supervising staff would not interfere while this confusion was going on so that they observe how we handle the situation….This is because they used to respect us and not correct us within the group, but rather they used to put us aside and give us feedback*. (ID#: 1237, community health worker, female)

A parent peer (ID#: 1128, female) shared that the feedback from the supervising team allowed them to do better in the subsequent sessions. 

*They would also give us feedback when we delivered the sessions well and advise us accordingly so that we would do better the next time; we would eventually do well following the support from the supervising team.* (ID#: 1128, parent peer female)

## Discussion

In this study, we explored the experiences of parent peers and community health workers with the task-shifting of an evidence-based multiple family group intervention designed for children with behavioral challenges and their families in southwest Uganda. We examined the factors that contributed to their decision to participate in the program, their experience with the training they received as well as the delivery of the intervention to better understand the acceptability and appropriateness of task-shifting this intervention to this group of lay providers in the Uganda context.

Though there were some initial concerns, especially around the lack of previous experience and the time commitment, all facilitators expressed an intrinsic motivation to participate in the program and be trained as facilitators. They believed that this intervention was highly relevant to the needs of the families in their communities, and they wanted to be part of it so that they could help their communities get better. This finding underscores the acceptability of task-shifting the evidence-based Amaka Amasanyufu intervention and mirrors other studies in sub-Saharan Africa where lay providers saw their new role as an opportunity to help their communities and achieve fulfillment [48–54]. A study on a family-based child mental health intervention in Kenya also reported similar findings [25].

Facilitators also mentioned that they thought the program would be relevant for their own family lives as well. This was a theme that came up later again when asked about their experiences with the training, where they mentioned that they learned skills and content that they applied with their family members, including children, and observed a positive change in their family relations. For some, this was a motivating factor for them to continue attending the training sessions. A similar finding was reported in a study that explored lay counselors’ experiences of task-shifting a family-based intervention in Kenya, where the counselors applied the skills learned to their own lives, including professional and volunteer roles. Wall and colleagues [25] attributed this to the helper therapy principle described by Riesman [55, 56] as lay providers, in this case parent peers and community health workers, also receiving benefits due to characteristics or problems similar to those receiving the intervention. In fact, given that Amaka Amasanyufu is a family strengthening intervention with concepts applicable to any family life (Rules, Relationships, Responsibility, Respectful communication − 4Rs; and Stress and Social support − 2Ss), it is not surprising that facilitators also benefited from what they learned during the training. As pointed out by Wall et al. [25] and others [50, 57, 58], facilitators’ ability to personally benefit from the intervention they are trained to deliver may increase retention, motivation, and effectiveness of interventions delivered by lay providers. Given their immediate applicability in one’s family life, family strengthening interventions may be particularly amenable to task-shifting.

Another motivation to agree to become an intervention facilitator was the financial incentives provided by the program. These incentives were also mentioned later as a factor that enabled them to attend the training. This was perceived by some as an expression of how much the team valued them as individuals participating in the program. In addition to making it easy for the facilitators to come to the sessions without worrying about the financial burden of the commute, the incentives allowed some facilitators to meet the needs of their families. Remuneration, or lack thereof, has been discussed as a significant issue among lay providers in the task-shifting literature. Cataldo and colleagues [21] found that lack of remuneration was a problem among home-based caregivers in HIV medication delivery in Zambia, despite their pride in developing technical care abilities. Similarly, in a study conducted by Smith and colleagues [59], community health workers in Malawi reported receiving low remuneration.

As discussed by Greenspan and colleagues [50], the options for remuneration for community health workers who have been extensively relied upon in task-shifting, range from regular salary to no compensation. While no salary for parent peers and community health workers has been provided in our study, they all received transport refunds to attend the training as well as during the 16-session intervention delivery -even though this has not been mentioned by the facilitators who participated in our qualitative interviews. While this has cost implications for programming, it is essential to carefully consider a contextually-relevant compensation plan, ideally in collaboration with lay provider input [25].

An initial concern in program participation expressed by facilitators, especially parent peers, was their lack of experience. However, they felt that the training content was not only relevant to the families in their communities and to them as caregivers, but also prepared them well for intervention delivery. In addition to the knowledge of the main constructs the intervention builds on, the training’s focus on facilitation skills was well-received. They also appreciated the role plays that allowed them to practice those skills and knowledge. Similar to findings reported by Wall et al. [25], facilitators felt they were ready and well-equipped to deliver the intervention at the end of the training. Even though they were still nervous at the beginning of the intervention delivery, they mentioned consistently using the knowledge and the various skills they learned during the sessions, especially when they struggled with actively engaging the families in the intervention. Adequate training has been identified as an important factor in task-shifting in other studies as well [23, 24, 59].

As documented by Wall and colleagues [25], some facilitators also mentioned time both as an initial concern and a challenge during the training. However, they said that given that they committed to the program, they made arrangements that would allow them to attend the training. This was an interesting finding that also was mentioned as one of the reasons why they continued attending the training. Even though they were told during consenting that they could withdraw at any time, some had the intrinsic motivation to honor their word.

Facilitators reported that they enjoyed delivering the intervention. Particularly, they enjoyed seeing the families engaged and participating actively in the sessions as well as seeing positive changes in the families, all of which are documented to be indicators of high perceived acceptability [18]. Some facilitators perceived this as a reflection of their sense of worthiness and competence, as also found in Bocoum et al.’s study [60] focused on task-shifting HIV-related services in Burkina Faso. In addition, some of the facilitators mentioned that they gained a respectable position within their communities as a result of becoming a facilitator in the program. The increased respect and status in the community because of their new role has also been documented in other studies examining community health workers’ experiences in sub-Saharan Africa [23, 25, 48, 53, 54, 61].

The challenges experienced during intervention delivery were related to difficulties with family attendance and engagement in sessions. However, community health workers and parent peers used the skills they learned during the training as well as one-on-one discussions with family members to cope with these issues, which for the most part, they reported were effective. Some of the facilitators also mentioned struggling with their co-facilitators, which in some cases required the training team to step in. Hence, the training received prior to intervention delivery may require a more explicit discussion on the dynamics of co-facilitation

The facilitators experienced an increased sense of self-efficacy and competence as the sessions progressed. Even though they felt the training prepared them well for intervention delivery, many reported being nervous at the beginning. As time went on, they felt they mastered how to effectively deliver the sessions. One of the factors that helped them along the way was supervision. Supervision, or lack thereof, is widely discussed in task-shifting literature [23, 49]. For instance, a recent qualitative systematic review showed that the experiences of lay health workers trained in task-shifting psychological interventions showed that lay health workers wanted more robust supervision [24]. Similarly, Ochieng and colleagues’ study [23] among community healthcare workers in Kenya found that appropriate supervision was one of the critical motivating factors to effectively perform their tasks. In our study, both parent peers and community health workers expressed great satisfaction with the supervision they received. While they first found it intimidating to have the supervisors in the session, they were very appreciative of the constructive feedback they received and the subtle and respectful ways in which the feedback was conveyed. They expressed that they had become better facilitators as a result of the supervision. Hence, despite its intensity and the time commitment it requires on the training team, it is critical to provide ongoing -and if possible immediate- supervision, especially during the first few rounds of intervention delivery.

In sum, community health workers and parent peers had a positive experience with the task-shifting of the evidence-based family strengthening intervention geared towards children experiencing behavioral challenges and their families. Their motivations to participate and remain engaged in the intervention as well as their readiness and enthusiasm to deliver the intervention were closely tied to intervention characteristics such as the relevance of the intervention content, financial incentives, strong training, and ongoing quality supervision. Facilitators’ positive experiences with the training, intervention delivery, and supervision are signs of high acceptability and appropriateness of task-shifting this intervention [18, 24], which are facilitator-level factors that may positively impact the quality and effectiveness of interventions [18], as hypothesized by the PRISM framework [20, 41].

It is particularly promising that both community health workers and parent peers had similar experiences. In fact, results from our randomized clinical trial show that among children with elevated symptoms of disruptive behavior disorders, Amaka Amasanyufu intervention was efficacious in reducing oppositional defiant disorder and impaired functioning relative to usual care. More importantly, there were no significant differences between the treatment group where the intervention was delivered by parent peers and the treatment group where it was delivered by community health workers [33].

The findings from this qualitative study, together with the results from the clinical trial, are particularly significant as they show that this intervention has the potential to be effectively task-shifted to not only community health workers -who are already extensively relied upon task-shifting but also to parent peers who seem equally receptive to being trained and deliver the intervention as well as equally effective in delivering it. Given the shortage of mental health professionals in sub-Saharan Africa, the possibility of expanding the lay counselor workforce in low-resource settings is particularly encouraging. However, child and adolescent mental health interventions interested in task-shifting should ensure that strong training and supervision, along with appropriate incentives, are provided to lay counselors. These interventions should also be able to identify and strengthen the motivations of their target workforce and be prepared to address their concerns.

### Limitations and future directions

The findings should be considered in the context of a few limitations. The qualitative interviews were cross-sectional, hence whether the facilitators maintained their position over time and/or continued to implement the intervention after the implementation of the project concluded remains to be further investigated. Relatedly, while the experiences of parent peers and community health workers mostly aligned across all study findings, the longer-term experiences may differ. Another limitation related to analysis is that we coded the transcripts in English after translation from Luganda, though the authors were able to ask for clarification where needed given that all interviews were first transcribed in the original language. While findings are limited to one specific intervention and geographical location, we provided a thick description of the study context, intervention, facilitator training, and study methods to enable other researchers to better assess the transferability of our study results to similar settings [47, 62] Hence, future studies should continue to examine the similarities and differences across contexts and types of mental health interventions. More research with parent peers as lay providers/counselors is also warranted given the scarcity of existing literature on that population.

## Conclusion

As the global mental health field continues to be interested in task-shifting interventions to lay providers, it is critical to better understand their experiences with the process. Community health workers and parent peers in this study reported positive experiences with the training, intervention delivery, and supervision as they prepared to deliver an evidence-based multiple family group family strengthening intervention for children with elevated symptoms of behavioral challenges and their families. They also identified both intrinsic and financial incentives that ensured their enrolment and attendance in the program. To ensure positive lay provider experiences that ultimately impact intervention success, successful examples should be studied closely so that evidence-based models can be put in place to support them through the process.

## Data Availability

The datasets analyzed during the current study are available from the corresponding author on reasonable request.

## Abbreviations

DBDs: Disruptive behavior disorders
LMICs: Low- and middle-income countries
MFG: Multiple family group
PTA: Parent-teacher association
SSA: Sub-Saharan Africa
VHTs: Village health teams
